# The relationship between childhood body weight and dental caries experience: an umbrella systematic review protocol

**DOI:** 10.1186/s13643-017-0610-8

**Published:** 2017-10-25

**Authors:** Susan J. Carson, Lamis Abuhaloob, Derek Richards, Mark P. Hector, Ruth Freeman

**Affiliations:** 1 Dental Health Services Research Unit, School of Dentistry, University of Dundee, Park Place, Dundee, DD1 4HR Scotland; 2 Dental Public Health, NHS Tayside, Dundee, Scotland; 3 Dental Public Health, NHS Forth Valley, Stirling, Scotland

**Keywords:** Dental public health, Child oral health, Dental caries, Body weight and umbrella systematic review

## Abstract

**Background:**

Obesity and dental caries are global public health problems which can impact in childhood and throughout the life course. In simple terms, childhood dental caries and body weight are linked via the common risk factor of diet. An association between dental caries and obesity has been described in a number of studies and reviews. However, similarly, a relationship has also been noted between low body weight and caries experience in children. This protocol will provide the framework for an umbrella review to address the following question: Does the available evidence support a relationship between dental caries experience and body weight in the child population?

**Methods:**

This review protocol outlines the process to carry out an umbrella systematic review which will synthesise previous reviews of childhood dental caries experience and body weight. An umbrella review methodology will be used to examine the methodological and reporting quality of existing reviews.

**Discussion:**

The final umbrella review aims to aggregate the available evidence in order to provide a summary for policymakers and to inform healthcare interventions.

**Systematic review registration:**

PROSPERO CRD42016047304

**Electronic supplementary material:**

The online version of this article (10.1186/s13643-017-0610-8) contains supplementary material, which is available to authorized users.

## Background

An increase in the number of individuals who are overweight or obese is proving to be one of the most challenging public health problems of the modern era [1]. Research suggests that there is a positive relationship between childhood obesity and obesity in adulthood [2, 3]. Dental caries (decay) in children is consistently found to be one of the most common non-communicable diseases worldwide [4, 5]. The Global Burden of Disease (GBD) 2010 Study found untreated dental caries in permanent teeth to be the most prevalent oral condition globally with around 35% of the population affected [5]. The consumption of excess free sugar has been implicated in the development of a number of non-communicable and chronic conditions such as obesity, type 2 diabetes and dental caries [6]. In simple terms, dental caries and body weight are linked via the shared risk factor of diet [7]. If free sugars are a common risk factor for both dental caries and body weight, what then is the relationship between dental caries and body weight?

An association between these two conditions have been reported in a number of individual studies; however, the results and methodologies are mixed and provide contradictory evidence [8–20]. The relationship between dental caries and body weight in children and adolescents has been looked at in three systematic reviews [21–23]. These reviews suggested that a relationship could exist, but that it is far from simplistic. The Hooley et al. review also found evidence which suggests that a converse, dental caries and low body weight relationship may exist [21]. In 2015, Public Health England released an evidence summary entitled “The relationship between dental caries and obesity in children” [24]. This made use of published literature and routine public health monitoring data to review and summarise what is known about the relationship between dental caries and obesity in individuals and populations. The review also found that much of the available evidence was in relation to the obesity and caries relationship in childhood. The results of this evidence summary describe four systematic reviews, the three detailed previously [21–23] and an additional paper by Kantovitz et al [25]. Again, an equivocal relationship between dental caries and body weight was found. The authors made use of the recently available ROBIS tool which found several of the reviews to be at high risk of bias [24, 26].

This wealth of varying quality and contradictory evidence suggests the need for an umbrella review to bring together the accessible evidence to permit an understanding of the relationship between dental caries and body weight in children in order to inform policy and potential healthcare intervention. It is expected that this work will be significant in defining the degree of adequacy in the methodological quality and reporting of systematic reviews of observational studies which look at the association between body weight and childhood caries experience.

### Objectives

The aim of this umbrella review is to summarise what is already known about the relationship between body weight and childhood dental caries experience in order to make recommendations for policy and to inform healthcare interventions which adopt the Common Risk Factor Approach (CRFA) [7].

### Research question

What is the relationship between dental caries experience and body weight in children?

## Methods

### Protocol development

This protocol has regarded for the Preferred Reporting Items for Systematic review and Meta-Analysis Protocols (PRISMA-P) checklist [27] and is registered with the PROSPERO database for systematic reviews [28]. A completed PRISMA-P checklist for the protocol is attached as an additional file (see Additional file 1). The protocol has been revised by the authors; all updates and amendments can be found within the PROSPERO record.

### Review methodology

In 2007, Moher et al. reported that “whilst systematic reviews are now produced in large numbers, the quality of their reporting is inconsistent” [29]. Given that a number of systematic reviews have been published on this topic, we will adopt a review of reviews or umbrella review method as described in Grand and Booth’s typology of reviews [30]. Umbrella review is the term applied to systematic reviews that draw together evidence from a series of other systematic reviews and meta-analyses [30, 31]. They are a means of reporting evidence other than that derived from randomised controlled trials and have been proposed as a way to provide a clear picture of a broad healthcare topic area [31–33]. The review will have regarded for the umbrella review methodology as outlined by Aromataris et al. in 2015 [31, 32].

This umbrella systematic review will specifically look at the quality of systematic reviews which have previously been carried out. It will appraise both the methodological and reporting characteristics of multiple reviews making use two available quality assessment tools: AMSTAR and PRISMA [29, 34]. In 2014, Pieper et al. tested the use of AMSTAR tool in appraising systematic reviews of non-randomised studies [35]. They found the tool to be reliable with only items 6 to 9 requiring further discussion between the reviewers. It was felt that this mainly arose due to the lack of standards for non-randomised studies when compared with randomised control trials, rather than an inherent problem with the questions themselves [35]. This review will focus on a quality assessment of the methodology and reporting of the reviews themselves, rather than the individual studies.

## Inclusion criteria

### Types of participants

Participants will be children defined as those under 18 years of age.

### Phenomena of interest

The phenomena of interest in terms of the relationship with dental caries experience are body weight. Body weight as measured by weight, BMI, waist circumference or any other recognised methods will be included.

### Outcomes

Outcomes for the primary, mixed and secondary dentition will be considered for inclusion. Any measure of dental caries including, but not limited to, DMFT (Decayed, Missing and Filled Teeth), DMFS (Decayed, Missing and Filled Surfaces), and caries incidence will be included.

### Context

No limitations will be made in relation to cultural factors such as geographic location, specific racial or gender-based interests.

### Types of studies

Studies will be systematic reviews or meta-analyses of observational studies in humans. Systematic reviews or meta-analyses which include cross-sectional, case series, case-control, cohort studies, or aetiology studies using data from an existing database will be included. All systematic reviews which look at the association between dental caries and body weight in children in the same individuals and populations will be considered.

A summary of the main study inclusion and exclusion criteria are provided in Table 1.

**Table 1:** Study inclusion and exclusion criteria

	Inclusion criteria	Exclusion criteria
Types of study	Systematic reviews and meta-analyses	All other types of study
Period of study	Any systematic review from 1990 to 2017	Systematic review prior to 1990
Types of participants	Children as defined by < 18 years	Studies where children are not the population of interest
Primary outcomes	Dental caries experience (as measured by any means)	Studies where dental caries is not the outcome of interest, i.e. other dental problems
Exposure/risk factor phenomena of interest	Body weight as measured by BMI, or any means	Studies which do not look at body weight in relation to dental caries experience
Language	Full articles in English	Relevant articles in languages other than English

### Review characteristics

Only full articles available in English will be reviewed. A list of possibly relevant titles in other languages will be provided as an appendix. As the availability and methodology of systematic reviews has increased since 1990 [33], it has been decided a priori to limit the search to systematic reviews from 1990 onwards.

## Search strategy

### Database search

The following electronic databases will be searched: MEDLINE, CINAHL, EMBASE, PsycINFO and Scopus. Additional systematic review databases, the JBI Database of Systematic Reviews and Implementation Reports, the Cochrane Register of Systematic Reviews and PROSPERO will also be searched. Manual screening of the reference lists of retrieved articles will also be carried out. A search for both black and unpublished grey literature will include databases which contain published reports from government and non-government organisations as well as additional material which have otherwise not been published [31, 36]. The Open Grey system for information on grey literature, the Agency for Healthcare Research and Quality database and the National Health and Medical Research Council database and Google Scholar will be used to aid in this search. The search process will be tailored to the specific host site.

### Search terms

We will conduct a search of electronic databases using MeSH terms and selected free-text terms (including those retrieved from published reviews which can be found in Table 2) as well as terms for systematic reviews and meta-analyses (Table 3). A search strategy has been drafted in collaboration with a Medical Librarian and has been trialled in MEDLINE; Additional file 2 shows this in more detail. This included planned limits so that the search could be repeated.

**Table 2:** Search terms retrieved from known reviews

Obesity, DMFT Index, tooth decay, waist circumference, skinfolds and body mass index. Operators OR or AND used [23].	
Obesity OR Overweight OR BMI OR body mass index OR weight AND caries OR dental caries OR oral health OR dmf OR teeth decay OR cavities AND child OR adol OR preschool OR toddlers OR pediat OR paediat. Included malnutrition OR malnourish [21].	
Keywords and index terms: obes, child, paediatric, weight, overweight, BMI, dental caries, primary dentition, dft, dmft, dmfs, dfs [22].

**Table 3:** Search concepts and keywords

Concept	MeSH terms	Title/abstract keywords
Dental caries	Dental Caries	caries, tooth decay
	Dental Health Surveys	–
	DMF Index	dmf, dmft, dmfs
	Oral Health	–
Body weight	Birth Weight	BMI
	Body Fat Distribution Anthropometry	
	Body Mass Index	
	Body Size	
	Body Weight	
	Body Weights and Measures Body Height	
	Waist Circumference	
Overweight	Obesity	obesity, over weight
	Paediatric Obesity	
	Obesity, Abdominal	
	Obesity, Morbid	
	Adiposity	
	Overweight	
Underweight systematic review	Thinness	underweight, low weight systematic review, meta-analysis
	–	
Child	Child	children, child, preschool
	Adolescent	
	Child, Preschool	
	Paediatrics	

### Study selection

The initial search will be carried out by the lead reviewer. Screening of titles and abstracts will be carried out independently by two reviewers. Any disagreement will be resolved by discussion and, if necessary, by a third reviewer. If a decision on inclusion cannot to be made based on the title or abstract, the full text will be considered and any reasons for exclusion which are based on this will be provided.

### Data extraction and management

Data from published reviews to be included will be abstracted into Endnote X7. Duplicates of individual systematic reviews will be deleted. A data extraction form has been designed to reflect the reporting standards for umbrella systematic reviews as outlined in the JBI methodology. Two reviewers will independently review the data, and any unresolved discrepancies will be settled through discussion with the review group. This tool is formed of the existing AMSTAR tool with additional reporting items from the PRISMA tool included [29, 34]. As this tool contains items derived from existing guidelines, it is believed the validity of the tool should be retained [34, 35]. The article flow and number of included studies will be described in a PRISMA flow diagram in the final report [29].

### Assessment of methodological quality

The AMSTAR [34] checklist will be used to assess the methodological quality of systematic reviews and meta-analyses that are eligible for inclusion. The methodological quality assessment will be carried out by two of the review team independently. Any unresolved discrepancies will be resolved through discussion and consensus agreement within the review group. The methodological quality of each retrieved review will be graded as good, fair or poor using the guidance criteria within the AMSTAR tool [34].

### Assessment of reporting bias

A risk of bias assessment is included within the AMSTAR tool [34]. If more than 10 studies with the same outcome measure are available for review, a funnel plot will be used to explore the potential of publication bias. Selective reporting within systematic reviews or of review findings will be recorded. This will be assessed through the comparison of actual reported outcomes with those stated within the review protocol.

### Reporting of findings

The PRISMA checklist will be used to assess the reporting quality of systematic reviews and meta-analyses that are eligible for inclusion [29]. The findings and conclusions from existing reviews will be presented in narrative form including tables and figures to aid in data presentation where appropriate. The limitations of each review will be reported and will include any confounders and potential moderating or mediating factors. It is anticipated that statistical pooling will not be possible due to variations in the outcome measure reporting. No subgroup analyses are planned; however, these will be presented as provided by the authors if appropriate. The whole umbrella review will be reported according to Preferred Reporting Items for Systematic reviews and Meta-Analyses (PRISMA) guidelines [29].

## Discussion

This review protocol outlines the process to carry out an umbrella systematic review which will look at existing reviews on childhood dental caries and body weight in order to compare and contrast their findings. The overall aim is to synthesise the available evidence in order to provide an updated summary for policymakers and to better inform healthcare interventions which make use of the CRFA.

## Additional files

Additional file 1: PRISMA-P checklist for protocol CRD42016047304 (version 2.1). PRISMA-P (Preferred Reporting Items for Systematic review and Meta-Analyses Protocols) 2015 checklist: recommended items to address in a systematic review protocol. (PDF 278 kb)

Additional file 2: Search strategy—MEDLINE (Ovid). Search strategy trialled in MEDLINE using EBSCO (search date: 06.10.16). (PDF 187 kb)

## Abbreviations

AMSTAR: A measurement tool to assess the methodological quality of systematic reviews
CRFA: Common Risk Factor Approach
DMFS: Decayed, Missing and Filled (tooth) Surfaces
DMFT: Decayed, Missing and Filled Teeth
GBD: Global Burden of Disease
MeSH: Medical Subject Headings
PRISMA: Preferred Reporting Items for Systematic review and Meta-Analyses
PRISMA-P: Preferred Reporting Items for Systematic review and Meta-Analysis Protocols
