# Supplementary material for: The relationship between childhood body weight and dental caries experience: an umbrella systematic review protocol
Source: Syst Rev. 2017 Oct 25;6:216. doi: 10.1186/s13643-017-0610-8 (PMC5657131; doi:10.1186/s13643-017-0610-8)
Supplement: Supplementary file 2 — Search strategy—MEDLINE (Ovid). Search strategy trialled in MEDLINE using EBSCO (search date: 06.10.16). (PDF 187 kb) [file 13643_2017_610_MOESM2_ESM.pdf]

Additional file 1: Search Strategy - Medline (Ovid)

The relationship between childhood body weight and dental caries experience: an umbrella systematic review protocol

Search strategy. Database: Medline using EBSCO (search date: 06.10.16)

Search	Query	Items Found
#1	Search (MH "Dental Caries+") OR "caries" OR "tooth decay" OR (MH "DMF Index") OR "dmf" OR "dmft" OR "dmfs" OR (MH "Oral Health") OR (MH "Dental Health Surveys")	69,233
#2	Search systematic review or meta-analysis	150,011
#3	Search ((MH "Dental Caries+") OR "caries" OR "tooth decay" OR (MH "DMF Index") OR "dmf" OR "dmft" OR "dmfs" OR (MH "Oral Health") OR (MH "Dental Health Surveys")) AND (#2)	601
#4	Search (MH "Body Weight+") OR (MH "Body Size+") OR (MH "Body Fat Distribution") OR (MH "Anthropometry") OR (MH "Body Weights and Measures") OR (MH "Birth Weight") OR (MH "Body Height") OR (MH "Waist Circumference") OR (MH "Body Mass Index") OR "BMI"	521,592
#5	Search (MH "Obesity") OR "obesity" OR (MH "Pediatric Obesity") OR (MH "Obesity, Abdominal") OR (MH "Obesity, Morbid") OR (MH "Adiposity") OR (MH "Overweight") OR "overweight"	262,483
#6	Search (MH "Thinness") OR "underweight" OR "low weight"	12,709
#7	Search (#4) OR (#5) OR (#6)	2,855,659

#8	Search (MH "Child+") OR "children" OR (MH "Adolescent") OR (MH "Child, Preschool") OR (MH "Pediatrics")	593,300
#9	Search (#3) AND (#7)	15
#10	Search (#9) AND (#8)	10
#11	Search (#10) AND Limiters (Date of Publication: 19900101-20151231)	10
#12	Search #11 AND Limiters (Date of Publication: 20060101-20151231; English Language)	9
